# Addition of silicon to boron foliar spray in cotton plants modulates the antioxidative system attenuating boron deficiency and toxicity

**DOI:** 10.1186/s12870-022-03721-7

**Published:** 2022-07-14

**Authors:** Jonas P. de Souza Júnior, Renato de M Prado, Cid N. S. Campos, Gilmar S. Sousa Junior, Kevein R. Oliveira, Jairo O. Cazetta, Priscila L. Gratão

**Affiliations:** 1 Faculty of Agricultural and Veterinarian Sciences. Department of Agricultural Production Sciences, São Paulo State University (UNESP), Jaboticabal, Via de acesso Prof. Paulo Donato Castellane, São Paulo, 14884900 Brazil; 2 Federal University of Mato Grosso Do Sul (UFMS), Rodovia MS 306, Km 105, Chapadão do Sul, Mato Grosso do Sul 79560-000 Brazil; 3 Faculty of Agricultural and Veterinarian Sciences. Department of Biology Applied to Agriculture, São Paulo State University (UNESP), Jaboticabal, Via de acesso Prof. Paulo Donato Castellane, São Paulo, 14884900 Brazil; 4 Institute of Plant Protection. Department of Integrated Plant Protection, Hungarian University of Agriculture and Life Sciences (MATE), Páter Károly utca. 1, Gödöllő, 2100 Hungary; 5 Faculty of Agricultural and Veterinarian Sciences. Department of Agricultural and Environmental Biotechnology, São Paulo State University (UNESP), Jaboticabal, Via de acesso Prof. Paulo Donato Castellane, São Paulo, 14884900 Brazil

**Keywords:** Beneficial element, Leaf spray, *Gossypium hirsutum* (cotton), Nutritional disorder, Non-enzymatic antioxidative mechanisms

## Abstract

**Background:**

Boron (B) nutritional disorders, either deficiency or toxicity, may lead to an increase in reactive oxygen species production, causing damage to cells. Oxidative damage in leaves can be attenuated by supplying silicon (Si). The aim of this study was to assess the effect of increasing foliar B accumulation on cotton plants to determine whether adding Si to the spray solution promotes gains to correct deficiency and toxicity of this micronutrient by decreasing oxidative stress via synthetizing proline and glycine-betaine, thereby raising dry matter production.

**Results:**

B deficiency or toxicity increased H_2_O_2_ and MDA leaf concentration in cotton plants. H_2_O_2_ and MDA leaf concentration declined, with quadratic adjustment, as a function of increased leaf B accumulation. Proline and glycine-betaine leaf concentration increased under B-deficiency and B-toxicity. In addition, production of these nonenzymatic antioxidant compounds was greater in plants under toxicity, in relation to deficient plants. Adding Si to the B spray solution reduced H_2_O_2_ and MDA concentration in the plants under nutrient deficiency or toxicity. Si reduced H_2_O_2_, primarily in B-deficient plants. Si also increased proline and glycine-betaine concentration, mainly in plants under B toxicity. Dry matter production of B-deficient cotton plants increased up to an application of 1.2 g L^− 1^ of B. The critical B level in the spray solution for deficiency and toxicity was observed at a concentration of 0.5 and 1.9 g L^− 1^ of B, respectively, in the presence of Si, and 0.4 and 1.9 g L^− 1^ of B without it. In addition, the presence of Si in the B solution raised dry matter production in all B concentrations evaluated in this study.

**Conclusion:**

Our findings demonstrated that adding Si to a B solution is important in the foliar spraying of cotton plants because it increases proline and glycine-betaine production and reduces H_2_O_2_ and MDA concentration, in addition to mitigating the oxidative stress in cotton plants under B deficiency or toxicity.

## Background

Boron (B) foliar spraying is a common practice performed by cotton (*Gossypium hirsutum* L.) growers in different regions worldwide due to B deficiency in different soils [1–3]. However, the difference between the adequate boron concentration and toxicity is small [1, 4, 5] and the toxic effect of this element can be verified in cotton plants when it is applied at high concentrations to leaves [3]. During the reproductive stage, plants have a higher B requirement compared to the vegetative stage [6], as in this stage, during the formation of pollen grains, pectins are used to form the cell wall and, in case of B deficiency, the rigidity of the cell wall is compromised [7]. Boron plays a role in pectin synthesis by forming cis-borate ester complexes that are obligatory compounds in cell wall constituents [6]. We believe that the cells in tissues generated in the formation of pollen grains have cells walls with a higher content of complexed boron. Hence, this logically increases B demand for the formation of cell walls. In addition, B deficiency decreases the transport of photoassimilates from leaves to flowers and fruits, impairing flower, seed and fruit formation [8]. Although B plays a vital role in plant reproduction [9], micronutrient deficiency can also impair the vegetative development of cotton plants [10]. This developmental impairment is due to the boron’s function in the cell wall structure, as well as due to its biological function in the metabolism of nucleic acids, proteins, phenols and in the functionality of the plasma membrane and sugar transport [11–14], cell elongation, and protein synthesis, resulting in increased cell division [15].

Thus, the adequate supply of boron for the cotton crop increases plant development and dry matter production [10]. However, excess B causes toxicity also decreasing the development of cotton plants [10, 15], which may cause metabolic disturbances in the formation of complexes with NAD^+^ in the ribosomes, affecting the formation of the RNA structure [14]. In addition, boron-related nutritional disorders (either deficiency or toxicity) may lead to an increase in reactive oxygen species (ROS) production, causing oxidative damage to cells, as observed in beets [16] and field peas [17]. Oxidative stress occurs when there is imbalance between ROS production and the antioxidant defense system (enzymatic and nonenzymatic), causing oxidative damage [18, 19]. However, the effects of boron-related nutritional disorder on oxidative stress in cotton plants remains unknown. Oxidative damage in leaves may be attenuated by supplying silicon (Si), which has been widely reported in cotton plants grown under abiotic stresses [20–22]. Si supply in cotton plants grown under stress reduces oxidative damage by decreasing the H_2_O_2_ content, lipid peroxidation and electrolyte leakage [22]. In addition, the beneficial element plays an important role in the modulation of antioxidant enzymes by increasing the transcription of genes involved in the defense response of plants, which has already been reported for several species [22–24]. The role played by B and Si in the enzymatic antioxidant system in leaves has been extensively explored and discussed [20–22]. Despite this, the non-enzymatic antioxidant system must be taken into consideration, and studies with this focus on cotton plants grown under B deficiency are scarce. In a review on B toxicity, Hua et al. [25] indicated that the non-enzymatic antioxidant system is important to regulate ROS neutralization in plant cells. Among non-enzymatic antioxidant compounds, the authors highlighted proline and glycine-betaine (GB). Proline is one of the most common osmolytes present in plants, being related to tolerance to various abiotic stresses [26] and to the neutralization of reactive oxygen species, attenuating the damage caused by lipid peroxidation [27]. Similar to proline, GB has an osmotic and antioxidant function related to its contribution to the maintenance of the structural and functional integrity of cells, which is possible due to the interaction between GB and the hydrophilic and hydrophobic domains of protein and membranous complexes in cells [28].

This raises an important question regarding the possibility of increasing the efficiency of B foliar sprayings in order to mitigate the harmful effects of deficiency or toxicity of this nutrients by including Si in the spray solution, although these elements must be chemically compatible. Therefore, the risk of Si polymerization in different nutrient solutions should also be taken into consideration, once this is the main disadvantage of using Si in the solution. The polymerization process of Si lead to the formation of insoluble silicon dioxide (SiO_2_), which compromises the benefit of this element to the plants. Silanol groups are formed during the polymerization and condensation of monosilicic acid. Then, the supersaturated acid solution is converted into its polymer form, forming insoluble spherical colloidal particles [29] that form long-chain networks, inducing visible changes in the solution from translucid to opaque or murky [30]. Si polymerization in aqueous solution is affected by the pH and Si concentration of the solution [30, 31], as well by the presence of stabilizer [32]. Stabilizers such as sorbitol help to stabilize Si monomers in solution, reducing the polymerization rate of this beneficial element and favoring its absorption [32], enabling Si addition to borate solutions for foliar application [30].

In this scenario, it is important to test the hypothesis that the a) deficiency and toxicity of B increases oxidative stress, with a consequent decrease in cotton plant development; and that b) Si addition to B solutions for foliar application, without polymerization, reduces the oxidative stress in cotton plants by increasing the production of proline and glycine-betaine. In this context, a study was conducted to evaluate the effect of increasing foliar B accumulation in the leaves of cotton plants to determine whether adding Si to the spray solution promotes gains to correct the deficiency and toxicity of this micronutrient by decreasing oxidative stress through the synthetization of proline and glycine-betaine, thereby increasing dry matter production.

## Results

### Control x factorial

Cotton plants grown without B deficiency (control) exhibited greater B accumulation, higher shoot dry matter production and lower B use efficiency when compared to those from the factorial treatment. In addition, Si accumulation, oxidative stress (measured by H_2_O_2_ and MDA production), and the induction of the proline nonenzymatic antioxidant system and GB systems were the lowest in control plants (Table 1).

**Table 1:** Analysis of variance between control and treatments for leaf B and Si accumulation, hydrogen peroxide (H_2_O_2_), malondialdehyde (MDA), proline, glycine-betaine, dry weight and B use efficiency in the shoots of B-deficient cotton plants

	Accumulation			
	Boron		Silicon	
	------------------- mg per plant -------------------			
**F-test**	257.1^**^		48.3^**^	
Control	754.1		6.9	
Factorial	376.2		14.1	
CV (%)	9.47		12.6	
	**H**_**2**_**O**_**2**_	**MDA**	**Proline**	**Glycine-betaine**
	μmol g^− 1^	g g^− 1^	μmol g^−1^	μg g^−1^
**F-test**	173.20^**^	198.87^**^	120.02^**^	12.59^**^
Control	328.36	2.03	23.33	4.54
Factorial	484.96	3.44	61.66	5.89
CV (%)	4.17	4.98	9.93	10.91
	**Dry weight**		**Boron use efficiency**	
	g		mg g^−1^	
**F-test**	78.82^**^		38.68^**^	
Control	6.50		0.04	
Factorial	4.78		0.10	
CV (%)	6.46		10.4	

^**^significant at 1% probability according to the F-test

### Turbidity test of the spray solution and B and Si accumulation in cotton plants leaves

**Fig. 1:**
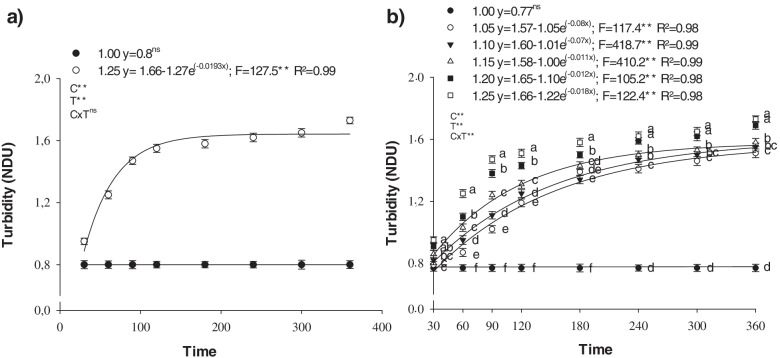
Turbidity index of the boron (2.5 g L^− 1^) + silicon (1.00 and 1.25 g L^− 1^) (**a**); and boron (2.5 g L^− 1^) + silicon mixture (1.00; 1.05; 1.10; 1.15; 1.20 and 1.25 g L^− 1^) as a function of time after solution preparation (**b**)

Adding 1.00 g L^− 1^ of Si in the form of SiKE to the B spray solution (2.50 g L^− 1^) did not increase the turbidity index of the solution, with an average value of 0.80 NDU (Fig. 1a). Adding 1.25 g L^− 1^ of Si to the B solution (2.50 g L^− 1^) caused an exponential rise in the turbidity index, reaching 1.73 NDU, 360 minutes after preparation (Fig. 1a). Increasing the Si concentration to 1.50 g L^− 1^ resulted in the immediate polymerization of the solution, precluding readings for turbidity index. Therefore, the ideal Si concentration in the spray solution was between 1.00 and 1.25 g L^− 1^, in order to avoid polymerization.

To improve Si concentration accuracy in the solution, a second assessment was carried out, indicating that the maximum amount of Si (SiKE) to be added to the B solution (2.50 g L^− 1^ of B) is 1.00 g L^− 1^ of Si, without changing the turbidity index, with an average value of 0.80 NDU (Fig. 1b). The increase in Si concentration from 1.05 to 1.25 g L^− 1^ raised the turbidity index exponentially, with a maximum of 1.51; 1.55; 1.58; 1.69 and 1.73 NDU after 360 minutes, for following Si concentrations 1.05; 1.10; 1.15; 1.20 and 1.25 g L^− 1^, respectively.

**Fig. 2:**
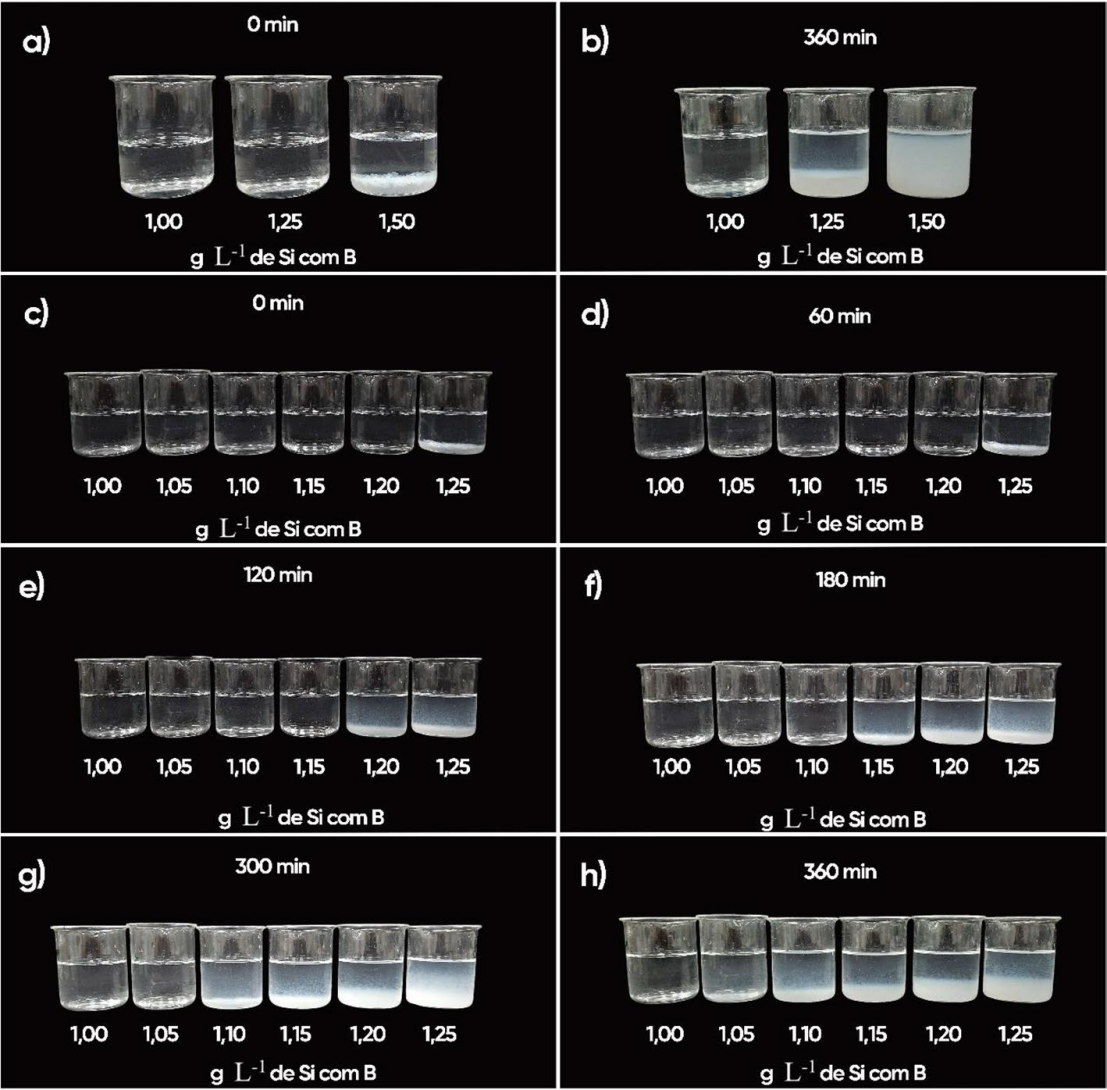
Assessment of the visual colorimetric change in the boron (2.50 g L^− 1^) + silicon mixture (1.00; 1.25 and 1.50 g L^− 1^) immediately (**a**) and 360 min after solution preparation (**b**); and boron (2.50 g L^− 1^) + silicon mixture (1.00; 1.05; 1.10; 1.15; 1.20 and 1.25 g L^− 1^) at 0 (c); 60 (d); 120 (e); 180 (f); 300 (g) and 360 (h) min after solution preparation

With respect to the solution color, the solution mixture with Si (1.00 g L^− 1^) and B (2.50 g L^− 1^) showed visible changes during the period assessed (Fig. 2). Adding Si (1.25 g L^− 1^) resulted in the formation of polymers immediately after solution preparation (Fig. 2a), evolving to a murky whitish color and gelatinous appearance after 360 minutes (Fig. 2b). Adding Si (1.50 g L^− 1^) to the B solution (2.50 g L^− 1^ of B) also resulted in polymerization, immediately forming a gel after solution preparation (Fig. 2a) and at 360 minutes (Fig. 2b).

The second turbidity index assessment, with a higher number of Si concentrations, showed no change in the color of the B spray solution (2.50 g L^− 1^) with the addition of Si between 1.00 and 1.25 g L^− 1^, except for polymer formation at a concentration of 1.25 g L^− 1^, in the first 60 min after solution preparation (Fig. 2c,d). At 120 minutes after B spray solution preparation (Fig. 2e), adding Si (1.25 and 1.20 g L^− 1^) to the B solution changed the color of the solution, evolving to a whitish color.

Gelatinization started 180 minutes (Fig. 2f) after spray solution preparation using the B solution mixed with Si (1.25 g L^− 1^); at 300 minutes (Fig. 2g) the B solution exhibited a gelatinous appearance when Si (1.20 g L^− 1^) was added, with a change in solution color with Si addition at the concentrations of 1.15 and 1.10 g L^− 1^.

At the end of the visual assessment, after 360 minutes (Fig. 2h) the B solution added with 1.25 and 1.20 g L^− 1^ of Si exhibited a thick gelatinous appearance. The solutions with 1.15 and 1.10 g L^− 1^ of Si showed a whitish color, and for those with 1.05 g L^− 1^ of Si it was possible to see a slight color change, indicating the onset of visible polymerization.

**Fig. 3:**
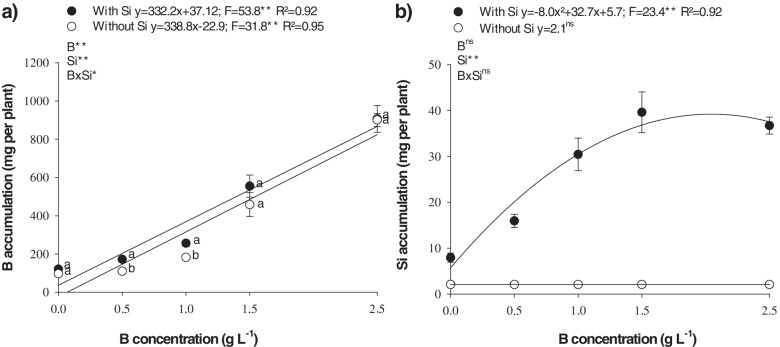
Shoot boron (**a**) and silicon accumulation (**b**) in B-deficient cotton plants as a function of different leaf boron (B) and silicon (Si) concentrations

Boron foliar spraying, in the absence or presence of Si (1.00 g L^− 1^), caused a linear increase in B accumulation in cotton plants shoots, reaching a maximum of 906.8 and 901.0 mg of B per plant, in the presence and absence of Si, respectively (Fig. 3a). In addition to that, at low B concentrations (0.5 and 1.00 g L^− 1^), adding Si to the B solution increased micronutrient absorption. Silicon accumulation in the leaves of cotton plants also increased with the increasing in B concentration, reaching 39,11 mg of Si per plant at 2,04 g L^− 1^ of B (Fig. 3b).

### Oxidative stress, proline and glycine-betaine

**Fig. 4:**
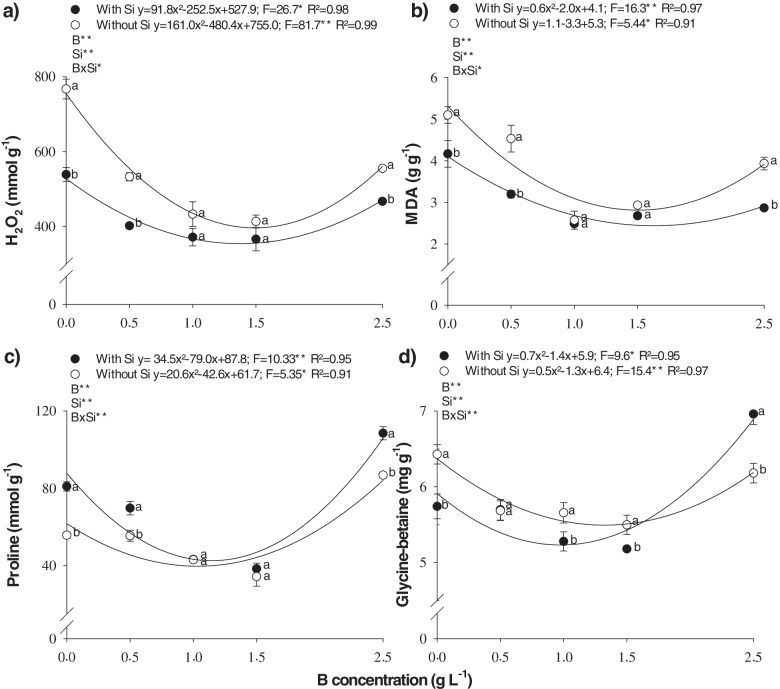
Hydrogen peroxie (H_2_O_2_) (**a**), malondialdehyde (MDA) (**b**), proline (**c**) and glycine-betaine (**d**) production in B-deficient cotton plants as a function of different leaf boron (B) and silicon (Si) concentrations

B deficiency (0.0 and 0.5 g L^− 1^ of B) or toxicity (2.5 g L^− 1^ of B) increased H_2_O_2_ (Fig. 4a) and MDA concentration (Fig. 4b).

**Fig. 5:**
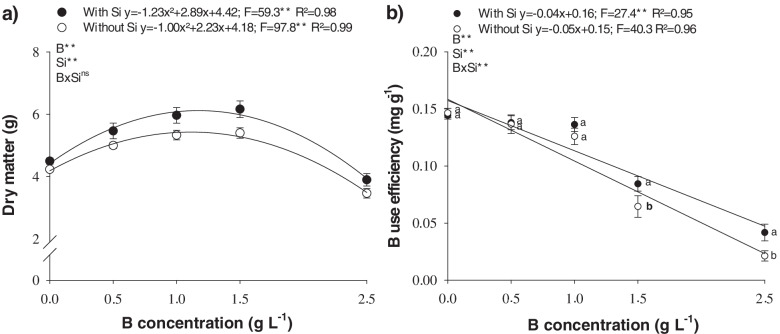
Shoot dry weight (**a**) and boron use efficiency (**b**) in B-deficient cotton plants as a function of different leaf boron (B) and silicon (Si) concentrations

H_2_O_2_ concentration declined as a function of increased leaf B accumulation, with a minimum point at B concentrations of 1.37 and 1.49 g L^− 1^ in the presence and absence of Si, respectively, (Fig. 4a); and B concentrations of 1.66 and 1.5 g L^− 1^ in the presence and absence of Si, respectively, for MDA (Fig. 4b). Boron deficiency stood out in H_2_O_2_ (Fig. 4a) and MDA production (Fig. 4b) when compared to its toxicity. In the 0.0 g L^− 1^ of B treatment, 539.1 and 767.3 μmol g^− 1^ of H_2_O_2_ was produced in the presence and absence of Si, respectively. These values represent an increase of 15 and 38% in H_2_O_2_ produced by plants grown under B toxicity (2.5 g L^− 1^), which obtained 467.3 and 555.4 μmol g^− 1^, in the presence and absence of Si, respectively (Fig. 5a). In the same treatment (0.0 g L^− 1^ of B), MDA concentration was 4.2 and 5.1 g g^− 1^, representing an increase of 50 and 30% compared to the 2.5 g L^− 1^ of B treatment, which produced 2.8 and 3.9 g g^− 1^ of MDA in the presence and absence of Si, respectively (Fig. 4b).

Proline (Fig. 4c) and glycine-betaine (Fig. 4d) concentration declined with a quadratic adjustment as a function of the rise in B concentration. The minimum points for proline were observed with the addition of 1.14 and 1.03 g L^− 1^ of B, and a production of 42.6 and 39.7 μmol g^− 1^ of proline, in the presence and absence of Si, respectively (Fig. 4c). The minimum points for glycine-betaine occurred with 1.0 and 1.3 g L^− 1^ of B, and a production of 5.2 and 5.5 g g^− 1^, in the presence and absence of Si, respectively (Fig. 4d).

In addition, production of these nonenzymatic antioxidant compounds was greater in plants under toxicity, with a proline concentration of 108.5 and 86.89 g g^− 1^, an increase of 34 and 56% in relation to deficient plants, which produced 81.0 and 55.8 g g^− 1^ in the presence and absence of Si, respectively (Fig. 4c). Glycine-betaine concentration was also higher in plants under toxicity, primarily in the presence of Si, with a production of 6.96 g g^− 1^ of glycine-betaine. This represents a 21% increase in relation to nutrient-deficient plants, which produced 5.74 g g^− 1^ of glycine-betaine (Fig. 4d). In the absence of Si, the increase was 4% in plants under toxicity (6.42 g g^− 1^) when compared to those with nutrient deficiency (6.18 g g^− 1^).

Adding Si to the B spray solution reduced H_2_O_2_ (Fig. 4a) and MDA concentration (Fig. 4b) in the plants under nutrient deficiency (0.0 and 0.5 g L^− 1^ of B) and those under toxicity (2.5 g L^− 1^ of B). Si reduced H_2_O_2_, primarily in B-deficient plants, with a production of 539.1 and 402.2 μmol g^− 1^ for 0.0 and 0.5 g L^− 1^ of B, respectively (Fig. 4a). These values are 29 and 24% lower compared to H_2_O_2_ production in the absence of the element, which was 767.3 and 532.9 μmol g^− 1^ for 0.0 and 0.5 g L^− 1^ of B, respectively (Fig. 4b). Si also increased proline (Fig. 4c) and glycine-betaine concentration (Fig. 4d), mainly in plants under B toxicity (2.5 g L^− 1^ of B).

### Dry matter production

Dry matter production in B-deficient cotton plants increased with B applications of 1.2 and 1.1 g L^− 1^, with maximum production of 6.1 and 5.4 g in the presence and absence of Si, respectively (Fig. 5a).

The critical B level in the spray solution was established in cotton plants for deficiency and toxicity, with a 10% decline in total dry matter produced at B concentrations of 0.5 and 1.9 g L^− 1^, respectively, in the presence of Si, and 0.4 and 1.9 g L^− 1^ of B without it. In addition, the presence of Si in the B solution raised dry matter production in all B concentrations evaluated in this study (Fig. 5a).

The efficiency of shoot B applications in the absence or presence of Si (Fig. 5b) caused a linear reduction with an increase in the B concentration applied. The presence of Si in the B solution raised B use efficiency at concentrations of 1.5 and 2.5 g L^− 1^.

## Discussion

Nutritional disorders are related to several losses in plant metabolism [6], and this effect occurs due to an increase in ROS (Table 1) in cotton plants, as also observed in beet [16] and field peas [17]. This effect is known for its B toxicity, being considered as the cause of oxidative damage to cells [25]. The adequate supply of B attenuates oxidative stress (Fig. 4a,b) by, first stimulating stress defense mechanisms [33]. Micronutrient deficiency, in turn, reduces the antioxidant system by decreasing the metabolic pathways for the biosynthesis of antioxidant compounds [13, 34] or by using the antioxidant system for other metabolic pathways.

A plant defense mechanism against B deficiency was evidenced in cotton grown under deficiency of this nutrient, as there was an increase in proline content (Fig. 4c). Proline has an important function in enhancing plant tolerance to abiotic stresses and reducing oxidative damages, since it increases the redox potential, which is essential to the antioxidant defense mechanism of plants, replenishing the supply of NADP^+^ [35]. NADP^+^ is generated during proline synthesis and its oxidation results in the production of NADPH, which acts as a buffering agent of the redox potential inside the cell [36] by binding to ROS and easily neutralizing them [37]. Moreover, plants under stressful conditions exhibit an increase in proline concentration, given the excess production of low molecular weight compounds that are highly soluble as compatible organic solutes.

Another important non-enzymatic antioxidant component is the GB content, which increased both B deficiency and B toxicity in cotton plants (Fig. 4d). This probably occurs as GB has an antioxidant effect, contributing to the maintenance of the structural integrity and to the functioning of cells, which is possible due to its interaction with the hydrophilic and hydrophobic domains of protein and membrane complex [28]. In addition, GB reduces ROS contents, contributing to their homeostasis [38] and inducing gene expression related to stress defense mechanisms through the octadecanoid pathway [39]. Therefore, this increase in GB activates several antioxidant mechanisms in cotton plants grown under stress (B deficiency and toxicity), which was verified by Hamani et al. [40], who observed that exogenous application of GB in this species under stress (saline) decreased oxidative stress.

Given this context, it is pertinent to highlight that the objective of this study was to evaluate the effect of increasing B accumulation in the leaves of cotton plants to determine whether adding Si to the spray solution promotes gains to correct the deficiency and toxicity of this micronutrient by decreasing oxidative stress through the synthetization of proline and glycine-betaine, thereby increasing dry matter production. The addition of Si to the borate solution contributes to reduce oxidative damage in cotton leaves (Fig. 4a, b), increasing both proline (Fig. 4c) and GB (Fig. 4d) under stress conditions caused by B-related nutritional disorders. It is important to note that, for Si to have a beneficial effect, there was no spray polymerization for foliar application, which is important as Souza Júnior et al. [32] indicated that polymerization is a limiting factor in the foliar application of Si. The evaluation of the turbidity index enabled to detect soluble changes in the silicon solution (Fig. 2) before the visible phase (Fig. 1). It was evident that even at a high B concentration (2.5 g L^− 1^), a maximum of 1.00 g L^− 1^ Si can be added to the B solution without significant polymerization. The stability of this silicon solution is partially explained by the stabilizing effect of the sorbitol present in the product to ensure successful Si foliar application [32]. This is because adding stabilizers is an efficient alternative to reduce the polymerization of the silicon solution, thereby increasing Si leaf absorption in several crops [17, 40, 41], including cotton [10, 30, 32].

The low polymerization of Si in the B solution (2.50 g L^− 1^ B) can be confirmed in the present study by the efficiency of B foliar spraying, which was evidenced by the increased B and Si accumulation in the shoots of cotton plants (Fig. 3). Sorbitol has another effect in Si leaf absorption, since it decreases the deliquescence point of the drops deposited onto the leaf surface, thereby lowering the rate of water evaporation (Kubicki and Heaney, 2003), and favoring leaf absorption [6].

Adding Si to the B solution reduced H_2_O_2_ (Fig. 4a) and MDA production (Fig. 4b). This beneficial effect of Si is demonstrated by its role in increasing the activity of the nonenzymatic defense system, such as increasing proline (Fig. 4c) and GB (Fig. 4d). Similar results have been reported by other authors, indicating that Si applications activate enzymatic defense mechanisms [22] and, especially their non-enzymatic counterparts [42], given the increased production of proline and GB due to the protective/activating function of Si. However, there are reports that there is a reduction in the production of proline [43–45] and GB [45] after the application of Si, thus decreasing cell damage due to the protective effect of Si. It is possible that these differences are caused by genotypic differences and/or by the activation of other metabolic pathways where these compounds are degraded to form new compounds with greater antioxidant power, although this needs to be proven.

In the present study, we showed that Si modulates the nonenzymatic antioxidant system, thereby attenuating nutritional stress and favoring an increase in the dry matter production of cotton plants grown under B deficiency or toxicity (Fig. 5a). Thus, the antioxidant role played by Si could explain its benefits in B-deficient cotton plants, as reported by Souza Júnior et al. [10] and Barros et al. [46], who attributed it to the increased chlorophyll concentration and photosynthetic efficiency.

It is known that the toxicity of a chemical element promoted by foliar application occurs relatively fast and that there are few strategies to reverse leaf damage [43]. Therefore, our findings showed that leaf damage can be mitigated by adding Si to the B solution during spraying, demonstrating that Si has a high potential in reducing oxidative stress. This likely favored the plant metabolism, thereby increasing its capacity to convert the micronutrient into biomass, since Si increased the B use efficiency at the highest concentrations applied to the leaves (Fig. 5b).

The present study revealed the relationship between B and Si in cotton plants by proposing foliar spraying in B-deficient areas at the critical concentration of 0.5 g L^− 1^ of B with the addition of 1.0 g L^− 1^ Si, and by demonstrating that a B concentration above 1.9 g L^− 1^ may be toxic to cotton plants. This information regarding the interaction of these elements for better efficiency in foliar spraying in cotton plants has global implications, given that several growing regions of this crop have reported B deficiency.

## Conclusions

In conclusion, adding Si to a B solution for foliar spraying in cotton plants increases proline and glycine-betaine production and reduces H_2_O_2_ and MDA concentration, mitigating the oxidative stress in cotton plants under B deficiency or toxicity.

## Methods

### Growing conditions

**Fig. 6:**
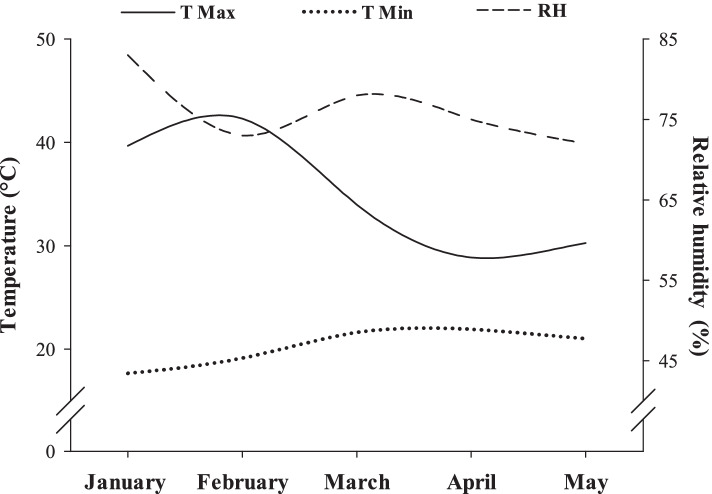
Maximum (T Max) and minimum temperature (T Min) and relative humidity (RH) in the greenhouse during the experimental period

The experiment was carried out between january and may (2019) in a greenhouse at the São Paulo State University (UNESP), Jaboticabal, Brazil. Seeds of cotton (*Gossypium hirsutum* L.) plants, cultivar 954 GLT, were used, commercially acquired from BASF, as it is a cultivar that presents consistent results in different regions and is highly adaptable to cotton producing regions in Brazil. Plants were grown in a soilless cultivation system. Relative humidity (78.4 ± 4.4%), maximum (35.1 ± 6.3 °C) and minimum temperature (22.0 ± 3.2 °C) were recorded with a thermometer hydrometer in the greenhouse during the entire experimental period (Fig. 6).

Cotton seeds were sown into trays containing sand previously washed with water and HCl solution (0.1 M). After germination, four seedlings were transplanted to plastic pots with a 7 dm^3^ capacity (upper diameter: 16 cm; lower diameter: 11 cm; height: 33 cm). Pots were filled with 6 dm^3^ of sand previously washed, as above. Thinning was performed with the plants in the vegetative growth stage F2 (two fully developed leaves), maintaining one plant per pot, which was considered as the experimental unit.

A complete nutrient solution was applied to the plants in the vegetative stage F4 (four fully developed leaves) [44]. The nutrient solution was prepared with Fe-EDDHMA as the iron source; pH between 5.5 and 6.5, adjusted with NaOH (1 M) or HCl (1 M) solution, and with a reduction only in B concentration (from 46.2 to 33.7 μmol L^− 1^), in order to cause moderate B deficiency in cotton plants grown in a soilless system [10].

The nutrient solution was applied for 1 days at 20% of its total concentration, as indicated by Hoagland and Arnon [44]. After this period, the concentration was increased to 40% for 1 week and then to 60%, which was maintained until the end of the experimental.

Pots were drained once a week to eliminate excess of salts, with 700 mL of deionized water being added to the substrate (sand) of each pot to drain the nutrient solution, which was then discarded. After 2 hours, a new nutrient solution was provided to the plants. This was performed for the entire duration of the experiment (rest of the growth cycle), with the nutrients required for plant development being regularly supplied.

### Experimental design and treatments

The experiment was carried out under a completely randomized block design (5 × 2 + 1) with four repetitions. The following treatments were applied: five foliar B concentrations (0.0; 0.5; 1.0; 1.5 and 2.5 g L^− 1^), absence and presence of Si (1.00 g L^− 1^); and one control treatment with no micronutrient deficiency, adding 46.2 μmol L^− 1^ of B to the nutrient solution during the entire experimental period.

Boron concentrations in the spray solution were 0%; 33%; 66%; 100 and 166% of the concentration recommended by Görmüs [45], where the author indicates B foliar spraying of 1.5 g L^− 1^ for cotton plants grown in a micronutrient-deficient environment.

The Si concentration to be mixed with the B solution and to be applied itself was defined based on the visual evaluation of the final solution to avoid using it with evidence of polymerization, which was measured by using the turbidity index.

### Turbidity index of the B + Si spray solution

To prepare the solution, boric acid (B: 175 g kg^− 1^; density: 1.43 g cm^− 3^; solubility in water at 20 °C: 47.2 g L^− 1^) was used as the B source, with the pH of the B spray solution adjusted to 9.0 using an NaOH solution (1 M). The Si source consisted of potassium silicate stabilized with sorbitol (SiKE; Si: 115 g L^− 1^; K_2_O: 113.85 g L^− 1^; sorbitol: 100 mL L^− 1^; pH:12.0). A potassium solution was also prepared with 47 mg L^− 1^ of K, in the form of KCl, to balance the macronutrient between treatments, since the Si source used in this study contained potassium.

Two turbidity index assessments of the B + Si spray solution were performed to determine the homogeneity of the Si solution, since an increase in its value would most likely increase the chance of Si polymerization. For this test, the B concentration of the solution was fixed at 2.5 g L^− 1^, the highest concentration used in this experiment. Assessment I consisted of adding Si to the B solution at concentrations of 1.00; 1.25 and 1.50 g L^− 1^ of Si; assessment II used a B mixture (2.5 g L^− 1^) with smaller intervals between Si concentrations (1.00; 1.05; 1.10; 1.15; 1.20 and 1.25 g L^− 1^) in order to increase the accuracy of the concentration in the spray solution. In both assessments, SiKE was used as Si source and H_3_BO_4_ as B source in the solution with a final volume of 50 mL, and pH adjusted to 9.05 ± 0.02 using a solution of HCl (1 M) or NaOH (1 M) at a temperature of 20 °C, with three repetitions. After the spray solution was prepared, the turbidity index was measured with a Tecnopon® microprocessed turbidity meter (model TB1000) at 0; 30; 60; 90; 120; 180; 240; 300 and 360 minutes, and photographs were taken using a camera with 9238 × 6928-pixel resolution.

All beakers with the different solutions were placed in front of a completely black background and were then photographed. The images were adjusted using Adobe Photoshop® in order to improve the contrast with the black background without changing B + Si solution color.

### Foliar application in the treatments

The solution was prepared according to the different treatments and immediately applied to the plants with a manual sprayer until run-off, in order to cover the entire plant shoot (all leaves). A 5.0 mL volume of the solution was applied to each pot. Sprayings occurred between 7 and 8 am, starting with cotton plants in the reproductive stage B1 (first completely developed flower bud), with four foliar applications 4 days apart. Temperature and relative humidity were measured by a thermometer hydrometer during applications, with temperature ranging between 20 and 23 °C, and relative humidity above 85%, conditions favorable for foliar spraying [6].

During foliar applications, all pots were covered with cotton to avoid any dropping or spillage from the plant shoot after being sprayed with the B + Si solution to the substrate (sand), in order to guarantee that B and Si elements absorption was totally foliar.

### Non-enzymatic antioxidant system (proline and glycine-betaine)

Leaves were collected 3 weeks after the B + Si mixture foliar application, being immediately frozen in liquid nitrogen and then placed in a freezer at − 80 °C to further assessment of the oxidative stress.

The rest of the shoot (branches and stem) was also collected, washed with water, detergent solution (0.1%), HCl solution (0.1%) and deionized water, being then placed in a forced air circulation oven at 65 ± 5 °C until constant mass. Further, branches and stems samples were weighed, grinded in a Wiley mill and stored for subsequent assessments.

In order to assess the oxidative stress, malondialdehyde (MDA) and hydrogen peroxide (H_2_O_2_) concentrations were determined on the leaves.

Initially, 0.4 g of the frozen plant material was weighed and grinded with 20% (m/v) polyvinylpyrrolidone and 0.1% trichloroacetic acid (TCA). Samples were centrifuged at 11,000 rotations per minute at 4 °C for 10 minutes. The supernatant was separated into Eppendorf tubes containing a 20% TCA solution and 5% thiobarbituric (TBA) acid. All samples were then incubated in a water bath for 30 minutes at 95 °C, and transferred to an ice bath for 10 minutes to stop the reaction, being centrifuged again at 11,000 rotations per minute at 4 °C for 10 minutes. Next, the samples were read in a spectrophotometer at wavelengths between 535 and 600 nm and MDA calculated using an extinction coefficient of 1.55 10^− 5^ mol^− 1^ cm^− 1^ [42].

H_2_O_2_ concentration was determined by homogenizing the frozen grinded plant tissue with 0.1% TBA, followed by centrifugation at 11,000 rotations per minute at 4 °C for 10 minutes. The supernatant was also transferred to Eppendorf tubes containing a buffer solution (pH 7.5) and potassium iode and then incubated for 1 h in an ice bath. Spectrophotometric readings were conducted at 390 nm wavelength, in line with the methodology described by Alexieva et al. [47].

Proline concentration was determined using the method proposed by Bates et al. [48]. Plant material was defrosted at ambient temperature and 0.5 g of fresh matter was macerated in liquid nitrogen and subsequently 2 mL of sulfosalicylic acid was added, followed by adding more 8 mL of the same reagent. The grinded material was doubly filtered in a glass funnel using filter paper. After filtering, 1 mL of glacial acetic acid, 1 mL of ninhydrin acid and 1 mL of plant extract were pipetted into a glass test tube. The tubes were then agitated and placed in a water bath at 100 °C for 1 hour and then into an ice bath to stop the reaction. Toluene was added (2 mL), followed by a 20-second agitation. Spectrophotometric readings were performed at 520 nm (adding 0.5 mL of the supernatant in a quartz cuvette).

Glycine-betaine concentration was determined according to the methodology proposed by Grieve and Grattan [49]. For that, 1 g of frozen plant material was placed in paper bags and dried in a forced air circulation oven at 80 °C for 4 days, then manually grinded in a crucible. Extracts were prepared by adding 5 mL of deionized water to 0.125 g of the macerated material, which remained under agitation for 24 hours at 25 °C. The extracts were mixed at 1:1 with H_2_SO_4_ (2 M), then maintained in an ice bath for 1 hour to stop the reaction. Further, 0.1 mL of KI-I2 was added to the tubes, which were then agitated and kept at 4 °C for 16 hours. The KI-I2 solution was previously prepared by diluting 15.7 g of iodine and 20 g of K in 100 mL of distilled water. The tubes were then centrifuged at 3500 rotations per minute for 15 minutes at 0 °C. The supernatant was discarded, leaving periodate crystals, which were dissolved in 4.5 mL of 1,2-dichloroethane. Two hours and 30 minutes later absorbance was read at a wavelength of 365 nm in a Beckman DU 640 spectrophotometer and the glycine-betaine concentration was calculated.

### Nutritional analysis of B and Si accumulation and plant shoot dry matter production

After analysis of the oxidative stress and nonenzymatic antioxidant compounds, leaves were defrosted at ambient temperature and then washed with water, neutral detergent solution (0.1%), HCl solution (0.1%) and deionized water. After decontamination, leaves were placed in a forced air circulation oven at 65 ± 5 °C until constant mass and then weighed. Samples were then manually grinded in a crucible and mixed with the previously collected macerated plant shoot samples (branches and stems).

Shoot dry matter consisted of the leaf dry matter left over from dry matter analysis of branches and stems collected initially. After the plant material was mixed, B concentration was determined by a dry digestion of the samples, burning in a muffle furnace at 400 °C, followed by colorimetric reaction with azomethine-H and colorimetric spectrophotometric reading [50]. Si concentration was determined from an alkaline digestion of the plant material with H_2_O_2_ and NaOH in an oven at 90 °C for 4 h [51], followed by colorimetric reaction with ammonium molybdate in acid medium (oxalic acid and hydrochloric acid), being then determined by colorimetric spectrophotometric readings [52].

B and Si accumulation were calculated as the product of B or Si concentration and shoot dry matter. In addition, B use efficiency was calculated as the ratio between the square of plant shoot dry matter values and plant shoot B accumulation, as described by Siddiqi and Glass [53].

### Statistical analysis

The data obtained were submitted to analysis of variance (F-test), and when significant, to polynomial regression or exponential growth.

The exponential growth model was used to study polymerization. Singular models with one or two parameters were tested, and the best fit models applied (those with the highest regression coefficient at 5% using the T-test).

The other variables were submitted to polynomial regression, the linear and quadratic mathematical models tested, and those with the best fit were applied. The model selection criterion established was the magnitude of significant polynomial regression coefficients at 5% probability using the T-test. When significant, the maximum and minimum points were obtained by the derivation of equations.

Statistical analyses were conducted with the Sisvar® software [54] and all graphs were created with Sigmaplot®.

## Data Availability

The datasets generated and/or analyzed during the current study are available from the corresponding author upon request.

## Abbreviations

B: Boron
GB: glycine-betaine
H_2_O_2_: Hydrogen peroxide
H_3_BO_4_: Boric acid
K_2_O: Potassium oxide
KCl: Potassium chloride
MDA: Malondialdehyde
NaOH: Sodium hydroxide
ROS: Reactive oxygen species
Si: Silicon
SiKE: Potassium silicate stabilized with sorbitol
SiO_2_: Silicon dioxide
